# Evaluation of Plasma microRNA-222 as a Biomarker for Gastric Cancer

**DOI:** 10.3390/jcm14010098

**Published:** 2024-12-27

**Authors:** Kotaro Wakamatsu, Atsushi Maruyama, Shinichi Okazumi

**Affiliations:** 1 Department of Surgery, Toho University Sakura Medical Center, 564-1 Shimoshizu, Sakura 285-8741, Chiba, Japan; sokazumiyrsh@sakura.med.toho-u.ac.jp; 2 Department of Life Science and Technology, Institute of Science Tokyo, 4259 B-57 Nagatsuta-cho, Midori, Yokohama 226-8501, Kanagawa, Japan; maruyama.a.e304@m.isct.ac.jp

**Keywords:** micro RNAs, gastric cancer, mir-222

## Abstract

**Background:** The dysregulation of microRNAs (miRNAs) has been detected in patients with gastric cancer (GC), which inspired the use of miRNAs as a novel biomarker for GC. In this study, we investigated the previously reported miRNA dysfunction in cancer tissues as a potential plasma biomarker for GC using quantitative reverse transcriptase polymerase chain reaction (RT-PCR). **Methods:** The published miRNA abnormalities were searched in the microRNA Cancer Association Database. Plasma samples were collected from patients with GC (n = 26) and controls (n = 17). The sensitivity and specificity of polyadenylation RT-PCR (PA-RT) and stem-loop RT-PCR (SL-RT) were compared. Statistical comparisons between patients with GC and controls were performed to identify miRNA biomarkers, and correlation analyses between the threshold cycle (Ct) values of miRNAs and various blood biochemical parameters were performed to elucidate the confounding factors. **Results:** mir-17, mir-21, mir-31, mir-99b, mir-222, and U6 were selected. PA-RT showed greater sensitivity and lower specificity than SL-RT (PA-RT vs. SL-RT, mean Ct: 19.6 vs. 29.2; coefficient of variation: 0.42 vs. 0.10). Adopting SL-RT owing to its higher specificity, only mir-222 was significantly upregulated in patients with GC (GC vs. control, miRNA expression: 15.4 vs. 5.27, *p* = 0.0098). Regarding the correlation between blood biochemical parameters and cells with miRNA expression, mir-31 and mir-99b were correlated with blood urea nitrogen, mir-17, mir-21, and mir-99b were negatively correlated with platelets, and mir-21 was correlated with neutrophils. No obvious correlations were noted between mir-222 expression and blood parameters. Receiver operating characteristic (ROC) curve analysis indicated that mir-222 identified GC patients with a maximum area under the curve (0.73, 95% confidence interval 0.57–0.89). **Conclusions:** Plasma mir-222 was confirmed to be dysregulated in patients with GC, irrespective of blood biochemical parameters.

## 1. Introduction

Gastric cancer (GC) is the fifth most common type of cancer and the fourth leading cause of cancer-related deaths worldwide [1]. The 5-year net survival outcomes of GC are reported to be 70% according to the recently published Japanese nationwide statistics. However, among more advanced GCs, such as stage III and IV disease, the survival rates are 40% and 5%, respectively, indicating that GC is a difficult-to-treat cancer. Nevertheless, treatment outcomes have gradually improved in recent years with the development of a wide range of chemotherapeutic options [2,3]. Complete surgical resection following an early diagnosis remains the most effective therapy for GC. Therefore, developing reliable and practical biomarkers that enable the early detection of GC is highly desirable. Recently, circulating microRNAs (miRNAs) have emerged as novel early detection biomarkers for a range of cancers [4].

Circulating miRNAs can successfully discriminate between cancers and non-cancers with high accuracy [5,6]. In line with this, several miRNA types have been shown to be differentially expressed in the serum of patients with GC [7]. However, detecting plasma miRNAs is difficult because their concentration is lower than that in the cancer tissue. Therefore, miRNA abnormalities assessed in cancer tissues do not warrant abnormalities in plasma miRNA. Owing to their scarcity in plasma, the reliable and accurate analysis of miRNAs is a major issue. RT-PCR is the gold standard method for measuring miRNAs in plasma due to its high sensitivity and specificity. However, the RT-PCR results for plasma can be compromised by sample handling [8] and are influenced by the different RT-PCR methods applied [9].

In this study, two major RT-PCR methods (polyadenylation and stem-loop RT-PCR) were compared to assess their reliability for miRNA measurement. Then, previously reported miRNA abnormalities in GC cancer tissue were confirmed to also be evident in plasma samples using the RT-PCR method. Influences of biochemical parameter and blood cell on the RT-PCR results were analyzed to determine the suitability of plasma miRNA as a GC biomarker.

## 2. Materials and Methods

### 2.1. Search for Potential Plasma miRNA Biomarkers for GC Using a Web Application

**Figure 1:**
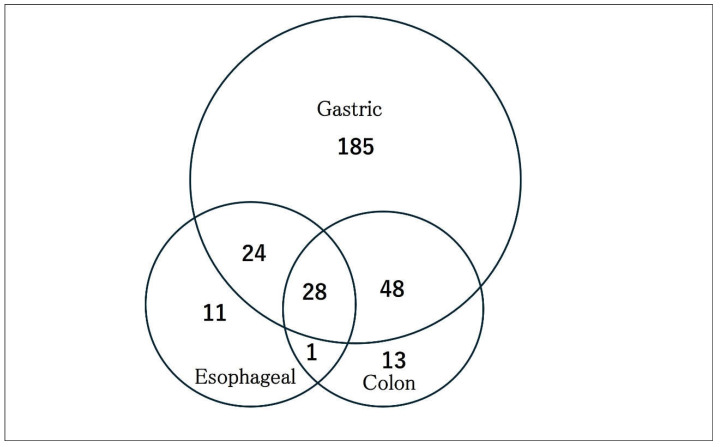
Venn diagram of the dysregulation of microRNAs (miRNAs). We searched the miRNA Cancer Association Database to identify miRNA abnormalities associated with gastric (GC), esophageal (EC), and colon cancer (CC). We found 285, 90, and 64 miRNA abnormalities in GC, CC, and EC, respectively. Overall, 185, 11, and 13 miRNA abnormalities were specific to GC, EC, and CC, respectively, and 28 miRNAs were dysfunctional in all three cancers.

A search for miRNA biomarker candidates using an interactive web application (“miRCancerDB”) which was established using the Cancer Genomic Atlas data, was performed [10]. The terms “Esophageal”, “Gastric”, and “Colon” were used to identify miRNA abnormalities for each cancer type. Overall, 66, 285, and 90 miRNAs were found to be dysregulated in esophageal, gastric, and colon cancer, respectively. Among them, 11, 185, and 13 miRNAs had miRNA abnormalities specific to esophageal, gastric, and colon cancer, respectively. We selected mir-99b as an esophageal cancer-specific biomarker and mir-17 and mir-222 as GC-specific biomarkers [11]. A total of 28 miRNAs were identified as being dysfunctional for the three cancer types, of which we selected mir-21 and mir-31. Some previous studies reported that mir-21 expression was upregulated in GC tissues, whereas others reported that mir-31 was downregulated [12] (Figure 1).

### 2.2. Patient Inclusion Criteria and Sample Preparation

miRNAs were prospectively purified from blood samples collected from patients visiting the hospital between April and June 2024. A total of 43 blood samples from patients with GC were collected into ethylenediaminetetraacetic acid-coated tubes and stored at 4 °C for 2–4 h until plasma separation. Patients with resectable GCs including no distant or peritoneal metastases underwent curative surgery. Blood samples from those patients were collected post operatively at first visit after discharge. A total of 17 patients were included in this group (“Control [CN]”). Patients with non-resectable GCs with distant or peritoneal metastasis received chemotherapy, and blood samples were collected before the first therapy session. A total of 26 patients were included in this group (“Gastric Cancer”). Before starting GC therapy, patients performance status was assessed, and those with an Eastern Cooperative Oncology Group (ECOG) performance status higher than 2 were excluded from the study.

The sample tube was centrifuged at 1750× *g* for 2 min at 4 °C to separate the plasma, which was stored at −80 °C until miRNA purification. The miRNA was purified from 300 μL of plasma using the Nucleospin miRNA Plasma kit (Marcherey-Nagel, Duren, Germany) according to the manufacturer’s instructions. This study was approved by the Institutional Review Board of Toho University (approval number: S22033, approval date: 30 March 2023), and informed consent was obtained from all participants. This study protocol adhered to the principles outlined in the Declaration of Helsinki from 1964 and later amendments. The following biochemical parameters were measured from the blood samples: albumin, alkaline phosphatase (ALP), alanine aminotransferase (ALT), aspartate aminotransferase (AST), blood urea nitrogen (BUN), creatinine, C-reactive protein (CRP), lactate dehydrogenase (LDH), γ-glutamyl transpeptidase (γGTP), and blood cell counts (white blood cells [WBCs], neutrophils, lymphocytes, monocytes, eosinophils, red blood cells [RBCs], and platelets). Tumor characteristics, including histological classification, depth of tumor invasion, lymph node metastasis, and distant metastasis, were determined using the Japanese Classification of Gastric Carcinoma [13].

### 2.3. miRNA Quantification Using Polyadenylation RT-PCR

miRNA was quantified via polyadenylation RT-PCR using Mir-X^TM^ miRNA First-Strand Synthesis and TB Green^®^ RT-PCR (Takara Bio USA Inc., Mountain View, CA, USA). For this, 2.0 μL of purified miRNA, 2.5 μL of imRQ buffer (2×), and 0.5 μL of mRQ enzyme were mixed to obtain a 5.0 μL reaction mixture. The reaction mixture was incubated in a PCR reaction tube for 60 min at 37 °C, 30 min at 85 °C, and 5 min at 85 °C, and subsequently held at 4 °C. Real-time PCR was performed using the StepOne Plus Real-Time PCR System (Thermo Fisher Scientific K.K, Tokyo, Japan). The 12.5 μL PCR reaction mixture contained 1 μL of the RT product, 6.25 μL of the TB Green Advantage Premix (2×), 0.25 μL of ROX dye (50×), 0.25 μL of the miRNA specific primer (10 μM), 0.25 μL of the mRQ3’ primer (10 μM), and 4.5 μL of nuclease-free distilled water. The reaction mixtures were incubated in a 96-well plate (MicroAmp Fast 96-well reaction plate 0.1 μL at 95 °C for 10 s, followed by 40 cycles at 95 °C for 5 s and at 60 °C for 20 s. Each sample was run in triplicate. The specific primer for measuring 99b-5p [5′CACCCGTAGAACCGACCTTGCG] was synthesized using FASMAC (Kanagawa, Atsugi, Japan). The primer for U6 was amplified using the Mir-X^TM^ miRNA First-Strand Synthesis kit.

### 2.4. miRNA Quantification Using Stem-Loop RT-PCR

miRNAs were quantified via stem-loop RT-PCR using TaqMan miRNA assays (Applied Biosystems, Foster City, CA, USA). A 7.5 μL reaction mixture was prepared by mixing 2.5 μL of purified miRNA, 0.5 μL of 10-mM dNTPs, 0.25 μL of MultiScribe^TM^ RT, 0.75 μL of 10× RT buffer, 0.10 μL of RNA inhibitor, 1.5 μL of 5× stem-loop RT primers (designed to specific miRNA targets), and 1.6 μL nuclease-free distilled water. The reaction mixture was incubated in a PCR reaction tube for 30 min at 16 °C, 30 min at 42 °C, and 5 min at 85 °C, and subsequently held at 4 °C. Real-time PCR was performed using a standard TaqMan PCR kit with the Step One Plus Real-Time PCR System. The 10 μL PCR reaction mixture contained 1 μL of the RT product, 1× TaqMan Universal PCR master mix, and 1× TaqMan miRNA primer. Reaction mixtures were incubated in a 96-well plate (MicroAmp Fast 96-well reaction plate [0.1 nL]) at 95 °C for 20 s, followed by 40 cycles at 95 °C for 1 s and 60 °C for 20 s. Each sample was run in triplicate. The purchased TaqMan miRNA assays were U6 snRNA (assay ID 001973), has-miR-17-5p (assay ID 002308), has-miR-21-5p (assay ID 000397), has-miR-99b-5p (assay ID 000436), and has-miR-222-3p (assay ID 002276) [14]. The threshold cycle (Ct) was used as the surrogate miRNA in the PCR. To calculate relative miRNA expression levels, the global standardization Ct(stand.) value was calculated by adding all miRNA values divided by the total sample number (n = 88; Ct(stand.) = 27.01). The relative miRNA expression was calculated as follows: relative miRNA expression = 2 exp − (Ct − Ct(stand.)).

### 2.5. Statistical Analysis

All statistical analyses were performed using the R project (version 2.3-0) with EZR on the R commander (version 1.35). Continuous variables are expressed as means ± standard deviation (SD) or medians with interquartile ranges, whereas categorical variables are expressed as numbers and percentages. Parametric data were compared between the groups using the unpaired Student’s *t*-test, whereas nonparametric data were analyzed using the Mann–Whitney U test. Categorical variables were compared using the Chi-squared test. Correlation analysis was performed by calculating Pearson’s correlation coefficients between the variables. The receiver operating characteristic (ROC) curve was used to determine GC using miRNA means and the area under the curve (AUC) was calculated with 95% confidence intervals (CIs). Statistical significance was defined as a two-sided *p*-value of <0.05.

## 3. Results

### 3.1. Comparison of Variability Between Polyadenylation and Stem-Loop RT-PCR for miRNA Quantification

mir-99b and U6 snRNA miRNA levels were measured by polyadenylation RT-PCR from 13 samples, and their relative Ct values were 24.3 ± 10.8 and 15.0 ± 6.11, respectively. The coefficients of variation (CV) were 0.44 and 0.41, respectively. Next, miRNA levels were measured by stem-loop RT-PCR from 43 samples. Their Ct values were 28.3 ± 3.03 and 30.1 ± 2.96, respectively, and their CVs were 0.11 and 0.09, respectively. The mean Ct values for polyadenylation and stem-loop RT-PCRs were 19.6 and 29.2, respectively, indicating that polyadenylation RT-PCR had higher sensitivity than stem-loop RT-PCR. The mean CV for polyadenylation RT-PCR was 0.42 and that for stem-loop RT-PCR was 0.10, indicating that stem-loop RT-PCR had higher specificity than polyadenylation RT-PCR. Based on these results, we adopted stem-loop RT-PCR for further study (Table 1).

### 3.2. Higher Plasma mir-222 Expression Was Noticed in Patients with Advanced Tumor Characteristics and a Lower Nutritional Status

miRNAs quantified using Ct values were compared between the GC and CN groups (n = 26 and n = 17, respectively). Only mir-222 expression significantly differed between the groups (GC vs. CN: 23.6 ± 2.92 vs. 25.8 ± 3.37; *p* = 0.0269). A comparison of relative miRNA expression showed that mir-222 and U6 expression significantly differed between the GC and CN groups (mir-222: 15.4 [7.79–45.0] vs. 5.27 [0.22–9.15], *p* = 0.0098; U6: 0.19 [0.084–0.454] vs. 0085 [0.0066–0.182], *p* = 0.0447). The least expressed miRNA was mir-31 (relative expression: ~0.01), and the most abundantly expressed miRNAs were mir-17 and mir-31 (relative expression: ~30). Furthermore, we compared demographic data and tumor characteristics between the groups. Age, sex ratio, weight, and height were comparable between the GC and CN groups (age: 70 ± 11.5 vs. 72.9 ± 11.5 years, *p* = 0.562; female/male: 15 [57.7%]/11 [42.3%] vs. 9 [52.9%]/8 [47.1%], *p* = 1.00; weight: 47.9 ± 8.8 vs. 50.7 ± 5.6 kg, *p* = 0.259; and height: 155 ± 8.0 vs. 159 ± 6.6 cm, *p* = 0.17). Although tumor pathology was similar, comprising a high rate of tubular adenocarcinoma (GC vs. CN, poorly differentiated/signet cell-type/tubular adenocarcinoma: 5 [19.2%]/4 [45.4%]/17 [65.4%] vs. 4 [23.5%]/0 [0%]/13 [76.5%], *p* = 0.236), the tumor characteristics significantly differed between the groups. Patients in the GC group had a higher number of invasive tumors (GC vs. CN, T1/T2/T3/T4: 0 [0%]/0 [0%]/14 [53.8%]/12 [46.2%] vs. 7 [41.2%]/3 [17.6%]/5 [2.94%]/5 [29.4%]/2 [11.8%], *p* < 0.01), multiple lymph node metastases (N0/N1/N2/N3: 14 [53.8%]/0 [0%]/2 [7.7%]/10 [38.5%] vs. 7 [41.2%]/2 [11.8%]/6 [35.3%]/2 [11.8%], *p* = 0.02,), and higher rates of distant metastasis (M0/M1: 5 [19.2%]/21 [80.8%] vs. 15 [88.2%]/2 [11.8%], *p* < 0.01) than those in the CN group. Moreover, lesions in the GC group tended to be more proximal than those in the CN group (esophageal junction (EGJ)/upper (U)/middle (M)/lower (L): 12 [46.2%]/2 [7.7%]/7 [26.9%]/5 [19.2%] vs. 5 [29.4%]/1 [5.9%]/1 [5.9%]/10 [58.8%], *p* = 0.04; Table 2). A comparison of biochemical parameters and blood cell counts showed that albumin levels and lymphocyte and platelet counts significantly differed between the groups (GC vs. CN, albumin: 3.3 [3.1–3.7] vs. 3.8 [3.6–3.8], *p* < 0.01; lymphocytes: 970 [816–1243] vs. 1871 [1367–2262], *p* < 0.01; platelets: 24.1 [13.1–28.3] vs. 31.9 [17.2–33.6], *p* = 0.03). Other parameters such as ALP, GPT, AST, BUN, creatinine, CRP, LDH, and γ-GTP, as well as neutrophil, monocyte, eosinophil, and RBC counts were comparable between the groups (Table 3).

### 3.3. Plasma miRNA Correlation Analysis

**Figure 2:**
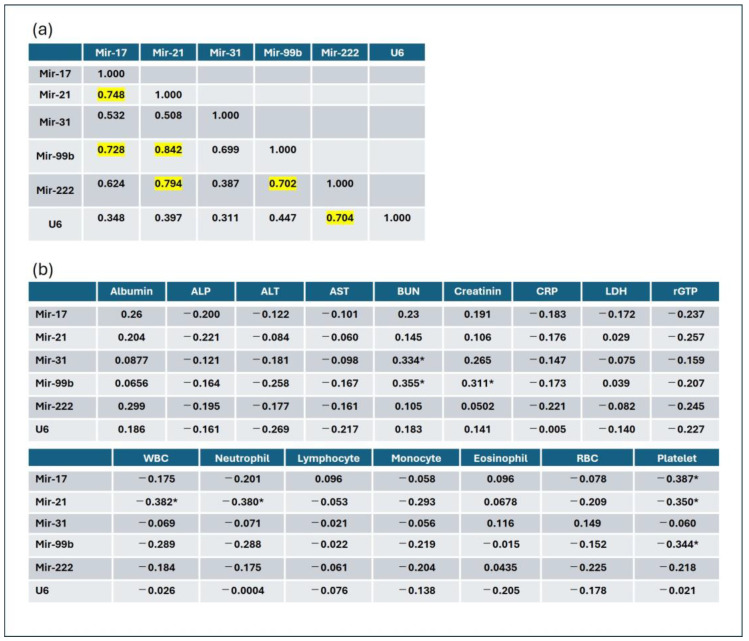
MicroRNA (miRNA) correlation analysis. (**a**) miRNA mutual correlation analysis was performed, and the correlation coefficient (r) was determined. Numbers marked by light-yellow indicate a strong correlation (r > 0.70). (**b**) Correlation analysis between the biochemical parameters and cell counts. The asterisk (*) indicates a significant correlation (*p* < 0.05).

The miRNA mutual correlation analysis showed that the Ct value of mir-17 was positively correlated with those of mir-21 and mir-99b (r = 0.748 and 0.728, respectively). The Ct value of mir-21 was positively correlated with those of mir-17, mir-99b, and mir-222 (r = 0.748, 0.842, and 0.794, respectively). The Ct value of mir-31 was not correlated with those of other miRNAs, and that of mir-99b was positively correlated with those of mir-17, mir-21, and mir-222 (r = 0.728, 0.842, and 0.702, respectively). The Ct value of mir-222 was strongly correlated with those of mir-21, mir-99b, and U6 (r = 0.794, 0.702, and 0.704, respectively). The Ct value of U6 was strongly correlated with that of mir-222 (r = 0.704). The Ct values of mir-17, mir-21, mir-99b, and mir-222 correlated with each other. However, the Ct value of mir-31 did not correlate with those of other miRNAs. Ct values of U6 and mir-222 correlated with each other. In addition, the correlation analysis of miRNA Ct values against biochemical parameters showed that the Ct value of mir-31 and BUN levels were weakly correlated (r = 0.334, *p* = 0.0285) and that the Ct value of mir-99b and BUN or creatinine levels were weakly correlated (r = 0.355, *p* = 0.195, and r = 0.311, *p* = 0.0424, respectively). Additionally, a correlation analysis of miRNA Ct values against blood cell counts was performed. Ct values of mir-17, mir-21, and mir-99b were negatively correlated with platelet counts (r = −0.387, *p* = 0.00103; r = −0.350, *p* = 0.0213; and r = −0.344, *p* = 0.0237, respectively). The Ct value of mir-21 was negatively correlated with the total WBC and neutrophil counts (r = −0.382, *p* = 0.0115, and r = −0.380, *p* = 0.012, respectively; Figure 2).

### 3.4. ROC Curve Analysis for GC Detection Using miRNA Expression

**Figure 3:**
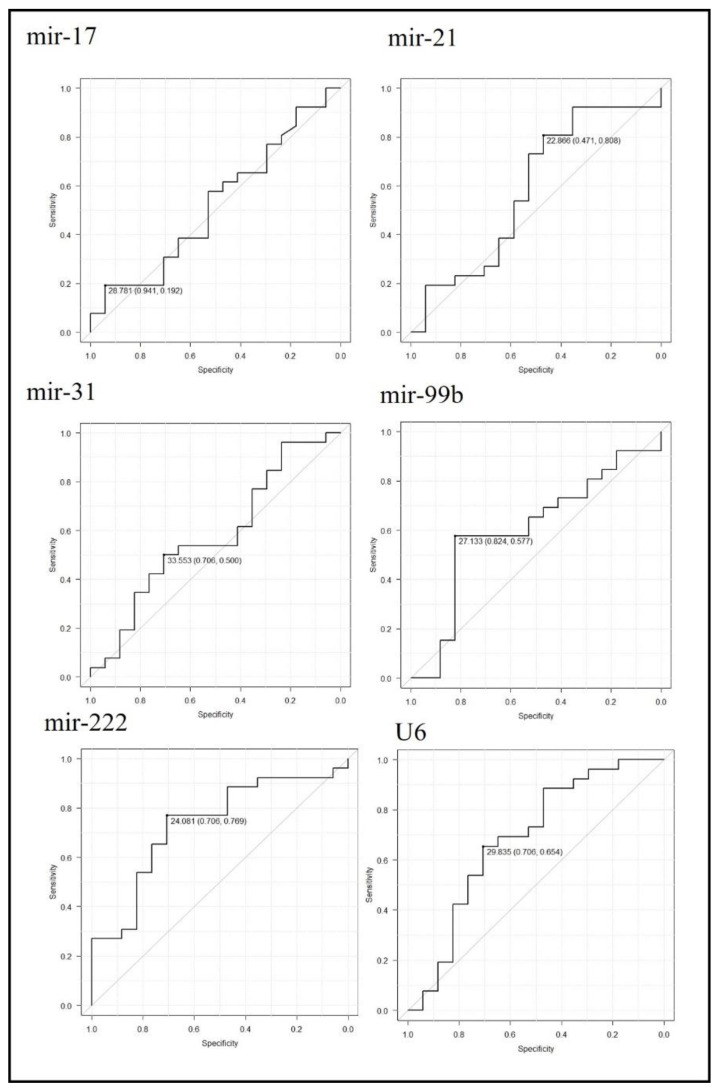
Receiver operating characteristic (ROC) curve analysis to detect gastric cancer patients using miRNA expression. The ROC curves corresponding to each miRNA Ct-value are shown. Cut-off Ct values with their specificity and sensitivity are indicated.

ROC curve analysis was performed to determine the best cut-off miRNA Ct values to detect GC. The cut-off values with maximum specificity and sensitivity were follows: mir-17, 27.7 (0.94, 0.19); mir-21, 22.8 (0.47, 0.80); mir-31, 33.5 (0.70, 0.50); mir-99b, 27.1 (0.82, 0.57); mir-222, 24.0 (0.70, 0.76); and U6, 29.8 (0.70,0. 65). AUCs with 95% CIs were also calculated: mir-17, 0.521 (0.33–0.70); mir-21, 0.584 (0.39–0.77); mir-31, 0.57 (0.39–0.761); mir-99b, 0.60 (0.42–0.78); mir-222, 0.73 (0.57–0.89); and U6, 0.68 (0.50–0.86). mir-222 was shown to be the most sensitive and specific miRNA to detect GC (Figure 3).

## 4. Discussion

Most of the previous miRNA abnormalities associated with GC were uncovered by researchers using microarray chips by comparing relatively small numbers of samples from cancer and normal tissue [12]. Therefore, abnormal plasma miRNA levels, which are lower compared to those present in cancer tissue, should be confirmed in order to be useful as clinical biomarkers. A recent study evaluated serum miRNA data from Japanese patients with cancer using machine learning, and demonstrated that the dysregulation of miRNA profiles could be used to identify various cancer types, irrespective of disease stage [15]. However, because of the small size of miRNAs and the sequence similarity between them, measuring miRNAs using microarrays is problematic for cross-hybridization. For a large-scale assessment using microarray platforms, the identified dysregulated miRNAs must be verified by quantitative RT-PCR, which is more sensitive and accurate compared to microarrays [16,17,18].

When the sensitivity and specificity of polyadenylation and stem-loop RT-PCRs were compared, polyadenylation RT-PCR showed greater sensitivity than stem-loop RT-PCR in terms of smaller Ct values for mir-99b and U6 [9]. Conversely, the accuracy of polyadenylation RT-PCR was lower than that of stem-loop RT-PCR method in terms of higher CV values. The stem-loop RT-PCR method includes additional nucleic acid target paring to detect RT products in the PCR phase, which is realized using TaqMan probes (dual-labeled probes with a fluorophore at the 5′ end and quenchers at the 3′ end). This unique characteristic could explain its higher specificity to detect miRNAs. Because miRNAs are short in length and similar in base composition, stem-loop RT-PCR with high specificity is an ideal and reliable miRNA detection method.

U6 is commonly used as an endogenous internal control to normalize miRNA expression in different samples. However, plasma U6 levels vary under certain conditions and are not suitable as internal controls for plasma miRNA measurements [19]. Therefore, global normalization, (calculated using all analyzed miRNA samples) was used to measure the relative plasma miRNA expression in this study. We found that mir-31, mir-99b, and U6 levels were relatively low (relative expression, <1) in plasma, whereas mir-17, mir-21, and mir-222 levels were high (relative expression, 5–45). Therefore, high plasma miRNA levels are required to ensure highly sensitive detection of a miRNA biomarker for GC. mir-17, mir-21, and mir-222 could therefore be considered as potential candidates for GC biomarkers.

This study revealed a higher plasma mi-222 expression in patients with GC. The upregulation of circulating mir-222 has been reported in previous studies, and mir-222 is thought to be an oncogenic miRNA [20,21,22]. Our findings confirm the previously reported mir-222 dysregulation. mir-222 targets, such as the reversion-inducing cysteine-rich protein with Kazal motifs [22], WEE1 [23], homeodomain interacting protein kinase 2 [24], vestigial-like family member 4 [25], and PTEN [26], modulate the proliferation, invasion, metastasis, and apoptosis of GC cells. Interestingly, using miRDB to predict mir-222 targets, 619 targets for mir-222-3p and 601 targets for mir-222-5p were found. Most of these mir-222 targets and their effects on GC have not yet been studied [27].

In contrast to mir-222, some plasma miRNAs are known to be downregulated in GC patients. mir-129-5p is one of these downregulated miRNAs, which has been intensively investigated [28]. Its targets have been reported to be IL-8 [29], COL1A1 [30], ADAM9 [31], HMGB1 [32], and SLC2A3 [33], and inducing mir-129-5p overexpression in tumor cells reduced malignant characteristics, including cell proliferation, migration, and invasion [29]. Although, plasma mir-222 expression levels had comparable power to detect GC according to the ROC curve analysis, the superiority of miRNAs as GC biomarkers over classical GC markers such as CEA and CA19-9 has not been previously reported [34]. To improve the sensitivity and specificity of miRNA as biomarkers, the combined use of upregulated and downregulated miRNAs would be a plausible strategy.

miRNA expression in GC tissue was not investigated in the present study, and therefore the origin of the plasma miRNA was not determined. However, miRNA mutual correlation analysis was performed to infer its origin. Since mir-222 expression was correlated with that of mir-21, mir-99b, and U6, these miRNAs were inferred to share the same origin. On the other hand, mir-31 expression did not correlate with that of other miRNAs, and therefore it was assumed to have an independent origin. Although mir-222 expression was known to be upregulated [20,21,22], measurement of miRNA in GC is necessary to identify the origin of plasma miRNAs.

The expressions of mir-17, mir-21, and mir-99b were positively correlated with platelet counts. Consistent with this finding, a previous study showed that residual platelets in plasma increase extracellular miRNA levels, and one freeze/thaw cycle of plasma dramatically increases extracellular miRNA levels by inducing miRNA release from platelets. This study suggests the importance of the remaining platelets as a source of contaminant miRNA, and the requirement for their meticulous removal from plasma samples before performing miRNA measurements for accurate quantification [35]. In contrast, the expression levels of mir-31, mir-222, and U6 were not correlated with platelet counts, suggesting that these miRNAs were less influenced by residual platelets in the plasma. Because it appears to be less influenced by platelet contamination, mir-222 is considered a suitable plasma tumor marker for GC.

In the context of plasma miRNA contamination, the effect of hemolysis should also be considered. High concentrations of several miRNAs were found in RBCs [36]. Despite the lack of a correlation between the Ct values of the analyzed miRNAs and RBC counts, mir-16 is one of the most abundant miRNAs in RBCs, and several studies have shown that its expression increases with the degree of hemolysis [37]. This result and those reported in previous studies suggest that miRNAs released from RBCs during sample handling could be an obstacle to accurate miRNA measurement. Therefore, miRNAs potentially affected by contamination derived from platelet degradation (such as mir-17, mir-21, and mir-99b) or RBC hemolysis (such as mir-16) are not ideal to be used as biomarkers. As no practical methods to differentiate miRNAs derived from blood cells and those derived from cancer cells have been described, it would be more appropriate to select as cancer biomarkers those miRNAs that are only released from cancer tissue.

In addition to platelets, mir-21 expression was correlated with neutrophil counts. Neutrophils, similar to platelets and RBCs, are a source of miRNAs, and specific miRNAs derived from neutrophils have been previously reported to function as regulators of inflammation [38]. mir-21 expression is associated with inflammatory diseases such as chronic obstructive pulmonary disease [39] and asthma [40]. In this study, we could not confirm mir-21 as a plasma biomarker for GC. However, as mir-21 expression increases with the neutrophil count, elucidating the possible role of mir-21 in inflammation and in GC progression is an intriguing research topic.

This study has several limitations. First, comparison of miRNA expression in the same patients before and after surgery was not performed. Second, other blood biochemistry parameters that could not be measured may have affected miRNA expression. Third, the origins of the miRNA were not determined, and they could only be presumed based on previous research. The current study pointed at mir-222 as a good potential biomarker for GS due to its significantly upregulated expression, sufficient quantity in plasma, and less chances of being affected by contamination from blood cells. To confirm mir-222 as a potentially valuable biomarker for GC, further research to overcome these limitations is required in the future.

## 5. Conclusions

The stem-loop RT-PCR method is more specific to detect plasma miRNA compared to the polyadenylation method. Among the miRNAs identified, mir-17, mir-21, mir-31, mir-99b, mir-222, and U6 were observed to be dysregulated in GC, but only mir-222 levels in plasma were high enough to differentiate GC. In addition, the expression of mir-17, mir-21, and mir-99b but not mir-222 were influenced by contamination with blood cells. The characteristics of mir-222 were particularly appropriate to be used as a potentially valuable biomarker to detect GC.

## Figures and Tables

**Table 1:** Comparison between polyadenylation and stem-loop RT-PCR methods.

Polyadenylation RT-PCR			
n = 13	Ct	SD	CV
mir-99b	24.3	10.8	0.44
U6	15	6.11	0.41
Mean	19.6	8.45	0.42
**Stem-loop RT-PCR**			
n = 43	Ct	SD	CV
mir-99b	28.3	3.03	0.11
U6	30.1	2.96	0.09
Mean	29.2	2.99	0.10

mir-99 and U6 levels were measured in 13 samples using polyadenylation RT-PCR and in 43 samples using stem-loop RT-PCR. The threshold cycle (Ct) values, standard deviations (SD), and coefficients of variation (CV) and their means are indicated in the table. RT-PCR, quantitative reverse transcription polymerase chain reaction.

**Table 2:** Comparisons of miRNA expressions and tumor characteristics between the GC and CN groups.

	GC (n = 26)	CN (n = 17)	*p*-Value
Micro RNAs			
Ct ^a^			
mir-17	22.8 ± 3.93	22.7 ± 3.95	0.92
mir-21	22.1 ± 2.91	23.3 ± 3.72	0.21
mir-31	33.7 ± 1.75	34.1 ± 2.13	0.46
mir-99b	28.0 ± 3.03	28.8 ± 3.06	0.38
mir-222	23.6 ± 2.92	25.8 ± 3.37	0.02 *
U6	29.4 ± 2.30	31.1 ± 3.57	0.05
Relative expression ^b^			
mir-17	34.2 [7.17–125]	40 [0.93–146]	0.82
mir-21	45.5 [23.5–10]	23.9 [0.86–112]	0.36
mir-31	0.01 [0.003–0.017]	0.008 [0.002–0.011]	0.39
mir-99b	1.02 [0.20–2.25]	0.30 [0.055–0.668]	0.26
mir-222	15.4 [7.79–45.0]	5.27 [0.22–9.15]	<0.01 **
U6	0.19 [0.084–0.454]	0.085 [0.0066–0.182]	0.04 *
Tumor characteristics			
Pathology ^c^			
Poorly differentiated	5 (19.2%)	4 (23.5%)	
Signet cell-type	4 (15.4%)	0 (0%)	
Tubular	17 (65.4%)	13 (76.5%)	0.23
Tumor thickness ^c^			
T1	0 (0%)	7 (41.2%)	
T2	0 (0%)	3 (17.6%)	
T3	14 (53.8%)	5 (29.4%)	
T4	12 (46.2%)	2 (11/8%)	<0.01 **
Lymph node metastasis ^c^			
N0	14 (53.8%)	7 (41.2%)	
N1	0 (0%)	2 (11.8%)	
N2	2 (7.7%)	6 (35.3%)	
N3	10 (38.5%)	2 (11.8%)	0.02 *
Distant metastasis ^c^			
M0	5 (19.2%)	15 (88.2%)	
M1	21 (80.8%)	2 (11.8%)	<0.01 **
Tumor location ^c^			
EGJ	12 (46.2%)	5 (29.4%)	
U	2 (7.7%)	1 (5.9%)	
M	7 (26.9%)	1 (5.9%)	
L	5 (19.2%)	10 (58.8%)	0.04 *

For statistical comparisons among patients in the gastric cancer (GC) and control (CN) groups, plasma miRNA levels are expressed as threshold cycle (Ct) values and relative expression levels, which are calculated using global standardization Ct values (Ct(stand.) = 27.0). Ct values are expressed as means ± standard deviation (SD). Relative expression levels are expressed as medians [interquartile range]. Tumor characteristics are described according to the Japanese Classification of Gastric Carcinoma (expressed in numbers [%]). ^a^ Student’s *t*-test, ^b^ Mann–Whitney U test, and ^c^ Chi-squared test are used. U, upper portion; M, middle portion; L, lower portion; EGJ, esophagogastric junction of the stomach. The *p*-values marked with * (<0.05) and ** (<0.01) are significant.

**Table 3:** Comparisons of biochemical parameters and blood cell counts between the GC and CN groups.

	GC (n = 26)	CN (n = 17)	*p*-Values
Biochemical parameters			
Albumin (g/dL)	3.3 [3.1–3.7]	3.8 [3.6–3.8]	<0.01 **
ALP (U/L)	86.5 [70.5–129]	80 [70–88]	0.48
ALT (IU/L)	22 [13.5–39]	13 [12.0–21]	0.25
AST (IU/L)	31.5 [17.2–41.2]	20 [15–30]	0.06
BUN (mg/dL)	15.1 [13.3–18.0]	16.9 [15.1–17.7]	0.54
Creatinine (mg/dL)	0.77 [0.65–0.83]	0.69 [0.55–0.81]	0.12
CRP (mg/dL)	0.095 [0.03–0.33]	0.05 [0.02–0.59]	0.63
LDH (U/L)	186 [152–213]	177 [170–194]	0.81
γGTP (IU/L)	23.5 [15.2–77]	29.0 [16–31]	0.58
Blood cells			
WBCs (/μL)	4235 [3577–5937]	5180 [3900–5660]	0.42
Neutrophils (/μL)	2812 [2166–4086]	2159 [1883–3615]	0.21
Lymphocytes (/μL)	970 [816–1243]	1871 [1367–2262]	<0.01 **
Monocytes (/μL)	334 [248–394]	308 [249–331]	0.63
Eosinophils (/μL)	145 [105–236]	129 [89–180]	0.34
RBCs (/μL)	355 [341–371]	392 [380–417]	0.18
Platelets (/μL)	24.1 [13.1–28.3]	31.9 [17.2–33.6]	0.03 *

Statistical comparisons (Mann–Whitney U test) of the biochemical parameters and blood cell counts between the gastric cancer (GC) and control (CN) groups were performed. Values are expressed as medians [interquartile range]. ALP, alkaline phosphatase; ALT, alanine aminotransferase; AST, aspartate aminotransferase; BUN, blood urea nitrogen; CRP, C-reactive protein; LDH, lactate dehydrogenase; γGTP, γ-glutamyl transpeptidase; WBC, white blood cell; RBC, red blood cell. *p*-values marked with * (<0.03) and ** (<0.01) are significant.

## Data Availability

The data that support the finding of this study are available on request from the corresponding author. The data are not publicly available due to their containing information that could compromise the privacy of research participants.
